# The cortical actin network regulates avidity-dependent binding of hyaluronan by the lymphatic vessel endothelial receptor LYVE-1

**DOI:** 10.1074/jbc.RA119.011992

**Published:** 2020-02-07

**Authors:** Tess A. Stanly, Marco Fritzsche, Suneale Banerji, Dilip Shrestha, Falk Schneider, Christian Eggeling, David G. Jackson

**Affiliations:** ‡ Medical Research Council Human Immunology Unit, University of Oxford, Oxford OX3 9DS, United Kingdom; § York Biomedical Research Institute, Department of Biology, University of York, York YO10 5DD, United Kingdom; ¶ Kennedy Institute for Rheumatology, University of Oxford, Oxford OX3 7FY, United Kingdom; ‖ Wolfson Imaging Centre, Weatherall Institute of Molecular Medicine, University of Oxford, Oxford OX3 9DS, United Kingdom; ** Leibniz Institute of Photonic Technology e.V., Albert-Einstein-Strasse 9, 07745 Jena, Germany; ‡‡ Institute of Applied Optics and Biophysics, Friedrich-Schiller-University Jena, Max-Wien Platz 4, 07743 Jena, Germany

**Keywords:** actin, fluorescence recovery after photobleaching (FRAP), fluorescence correlation spectroscopy (FCS), confocal microscopy, membrane biophysics, leukocyte, hyaluronan, endothelial cell, dendritic cell, receptor, stimulated emission depletion (STED) microscopy, immune system, membrane receptor dynamics, STED-FCS, lymphatic vessel endothelial hyaluronan receptor 1 (LYVE-1), sFCS

## Abstract

Lymphatic vessel endothelial hyaluronan receptor 1 (LYVE-1) mediates the docking and entry of dendritic cells to lymphatic vessels through selective adhesion to its ligand hyaluronan in the leukocyte surface glycocalyx. To bind hyaluronan efficiently, LYVE-1 must undergo surface clustering, a process that is induced efficiently by the large cross-linked assemblages of glycosaminoglycan present within leukocyte pericellular matrices but is induced poorly by the shorter polymer alone. These properties suggested that LYVE-1 may have limited mobility in the endothelial plasma membrane, but no biophysical investigation of these parameters has been carried out to date. Here, using super-resolution fluorescence microscopy and spectroscopy combined with biochemical analyses of the receptor in primary lymphatic endothelial cells, we provide the first evidence that LYVE-1 dynamics are indeed restricted by the submembranous actin network. We show that actin disruption not only increases LYVE-1 lateral diffusion but also enhances hyaluronan-binding activity. However, unlike the related leukocyte HA receptor CD44, which uses ERM and ankyrin motifs within its cytoplasmic tail to bind actin, LYVE-1 displays little if any direct interaction with actin, as determined by co-immunoprecipitation. Instead, as shown by super-resolution stimulated emission depletion microscopy in combination with fluorescence correlation spectroscopy, LYVE-1 diffusion is restricted by transient entrapment within submembranous actin corrals. These results point to an actin-mediated constraint on LYVE-1 clustering in lymphatic endothelium that tunes the receptor for selective engagement with hyaluronan assemblages in the glycocalyx that are large enough to cross-bridge the corral-bound LYVE-1 molecules and thereby facilitate leukocyte adhesion and transmigration.

## Introduction

The transmembrane glycoprotein LYVE-1^3^ is the principal receptor for the large pericellular matrix glycosaminoglycan hyaluronan (HA; (GlcNAcβ1–4GlcUAβ1–3)*_n_*) in lymphatic endothelium. Located in the distinctive overlapping junctions of initial lymphatic capillaries that serve as entry points for migrating leukocytes, LYVE-1 has been shown to play a key role in facilitating the docking and transit of antigen-presenting dendritic cells (DCs) (1) by engaging with HA present in the DC surface glycocalyx. Similar interactions between LYVE-1 and HA in pericellular matrix have also been shown to mediate the clearance of inflammatory macrophages via pericardial lymphatics during recovery from myocardial infarction (2) and the lymphatic dissemination of pathogenic strains of group A streptococci that bear a virulence-associated hyaluronan surface capsule (3).

How LYVE-1 mediates adhesion to the HA glycocalyx in these important physiological processes is unclear. Although HA polymers can be very large and in excess of 1 MDa, or 5,000 saccharides in length, each LYVE-1 homodimer interacts only with a short tract of some 15–20 sugar units and with relatively low binding affinity (*K_D_* = 8 μm) (4). Consequently, each polymer chain requires engagement with multiple receptors in tandem to achieve sufficient avidity for adhesion (5). Nevertheless, *in vitro* studies with primary lymphatic endothelial cells (LECs) have shown that even very high-molecular-weight HA polymers still bind poorly to LYVE-1 unless the receptor is first clustered using bivalent antibody, or alternatively the HA polymers are organized in higher-order multimers that can themselves induce LYVE-1 clustering (4, 6). Such findings have led us to postulate that the mobility of LYVE-1 may be limited in the endothelial plasma membrane, thus imposing a dependence on higher-order HA configurations to achieve the appropriate degree of receptor clustering (5). How the distribution and dynamics of LYVE-1 are controlled in the endothelial plasma membrane are, however, unknown.

A prerequisite for cluster-dependent ligand binding is the lateral mobility of the receptor in the plasma membrane (7–10), and one of the key cellular components influencing such mobility is the cortical actin cytoskeleton (11). This is evident in the case of prominent receptors, such as major histocompatibility complex class I and II (12, 13), interleukin-1 receptor α-subunit, transferrin receptor (14), FcϵRI (15, 16), CD1d (17), B-cell receptor–IgM, IgD, CD19 (18), natural killer cell receptors (19), and the leukocyte HA receptor CD44 (20–23). The cortical actin cytoskeleton consists of filaments (F-actin) that form a complex network in close contact with the cytoplasmic surface of the plasma membrane (<10–20 nm) (24). This network is highly dynamic, and the filaments are actively turned over by Arp2/3-mediated branching and formin-mediated extension, leading to changes in F-actin filament length, network mesh size, and cortex–membrane distance. Such processes occur rapidly in response to cell stimuli (*i.e.* between 1 and 10 s and 1 min) and can dramatically alter the structural integrity of the cell (16, 24–26). Moreover, they can transiently increase the lateral mobility of receptors in the plasma membrane by releasing them from confinement by the cortical actin meshwork, thereby altering their functional status (14, 16, 27–29). Whether the actin cytoskeleton influences LYVE-1 in such a manner has not yet been explored.

Here, we have used a combination of techniques, including flow cytometry, super-resolution stimulated emission depletion (STED) microscopy, fluorescence recovery after photobleaching (FRAP), and scanning as well as super-resolution STED fluorescence correlation spectroscopy (sFCS and STED-FCS, respectively), to probe the dynamics of LYVE-1 in primary human dermal lymphatic endothelial cells (HDLECs). Using such methodologies, we demonstrate that the lateral mobility of LYVE-1 in the plasma membrane is restricted by the underlying actin network and that its disruption leads to an increase in both LYVE-1 diffusion and, most importantly, HA binding. Additionally, we show this restricted diffusion is imposed not through direct physical interactions between actin and the LYVE-1 cytoplasmic tail, but rather by its entrapment within discrete submembrane actin corrals. Our findings reveal for the first time that native LYVE-1 is functionally compartmentalized in the endothelial plasma membrane and provide evidence that the actin cytoskeleton is an important dynamic regulator of LYVE-1 clustering during selective engagement of higher-order HA configurations, such as the leukocyte surface glycocalyx.

## Results

### Organization of LYVE-1 and F-actin in lymphatic endothelium

**Figure 1.**
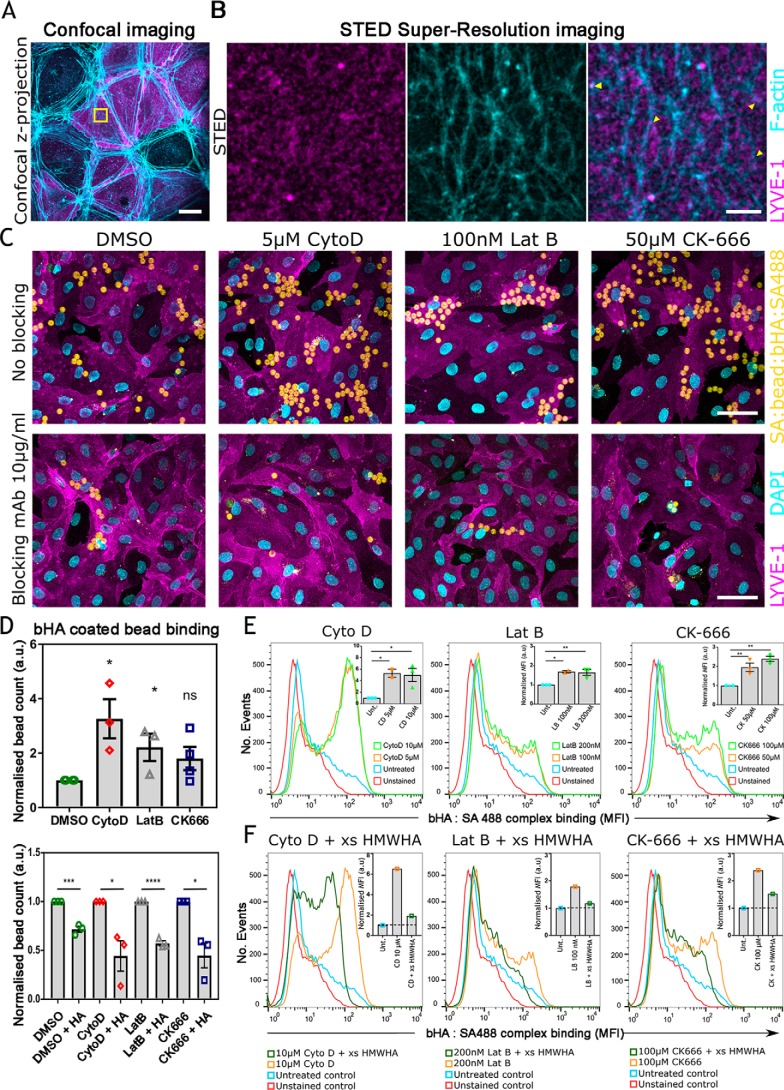
**Binding of HA to LYVE-1 in HDLECs is increased upon actin depolymerization.** *A*, confocal z-stack projection of hLYVE-1–transfected HDLECs, stained for LYVE-1 using LYVE-1 mAb/Alexa Fluor 594 conjugate (*magenta*) and for actin using Abberior STAR 635 phalloidin (*cyan*) (*scale bar*, 20 μm). *B*, super-resolution STED imaging of a 10 × 10-μm region of the HDLEC cell surface (*yellow boxed area* from *A*) to assess the localization of LYVE-1 (*magenta*) and actin (*cyan*). LYVE-1 was mostly located within corrals enclosed by the cortical actin meshwork and in a small number of cases appeared to overlie actin filaments themselves (*yellow arrowheads*) with a Pearson's correlation value of 0.1 ± 0.07. The *scale bar* (2 μm) applies to all three images shown. *C*, confocal z-stack projections showing binding of fluorescent bHA-coated beads (*yellow*) to monolayers of either DMSO (control)–, Cyto D–, Lat B–, or CK-666–treated HDLECs (*magenta*) either alone (*top*) or in the presence of a LYVE-1 HA blocking antibody (*bottom*). Images are representative of three experimental replicates. The *scale bar* (50 μm) applies to all eight images shown. Some of these images are also reproduced at higher magnification in Fig. S1 (see “Results”). *DAPI*, 4′,6-diamidino-2-phenylindole. *D*, quantification of bHA-coated bead binding from the data in *C* performed in the presence or absence of LYVE-1 HA-blocking antibody (*top* and *bottom panels*, respectively), indicating the sum from experimental replicates (10 fields/condition, *n* = 3 and *n* = 4 for CK-666 experimental replicated) with S.E. (*error bars*) and significance indicated by unpaired *t* test (Cyto D = 0.0129, Lat B = 0.0335, CK-666 = 0.1103; with blocking DMSO = 0.0007, Cyto D = 0.0229, Lat B = <0.0001, CK-666 = 0.0122). Blocking data are normalized to their respective nonblocking data. *E*, FACS histograms showing quantification of bHA:SA488 complex binding (*orange* and *green*) to HDLECs treated with either Cyto D, Lat B, or CK-666 or to untreated HDLEC controls (*blue*). *Insets*, median fluorescent intensity plots, normalized to untreated controls. Data are the mean ± S.E. from three replicate experiments. Statistics are compared using one-way analysis of variance with Dunnett's multiple comparison; *p* = 0.0211 for Cyto D, *p* = 0.0080 for Lat B, *p* = 0.0015 for CK-666. *F*, corresponding controls for the experiments in *E* performed in the presence or absence of excess unlabeled high-molecular-weight HA (*xs HMWHA*). *Insets*, median intensity plots normalized to the untreated conditions. Data are from two replicates. *,*p* ≤ 0.05; **, *p* ≤ 0.01; ***, *p* ≤ 0.001; ****, *p* ≤ 0.0001; *ns*, not significant.

To investigate the organization of LYVE-1 in relation to the actin cytoskeleton, we examined the relative distribution of both of these components in the plasma membrane of *in vitro* cultured primary HDLECs transfected with a full-length hLYVE-1 cDNA, using a combination of confocal and STED microscopy. Initial confocal imaging of the HDLEC monolayers was performed after fixation, permeabilization, and dual immunostaining for LYVE-1 (mAb 8C and secondary Alexa Fluor 594–labeled antibody) and F-actin (phalloidin Abberior® STAR 635). The results (Fig. 1*A*) revealed the actin cytoskeleton as a dense arrangement of fibers concentrated at the borders of each endothelial cell, whereas LYVE-1 had a more diffuse distribution, present mostly within the cell body. Although a proportion of the receptor appeared to partially co-localize with actin at cell:cell junctions, the relationship could not be reliably discerned due to the inherent limitations of conventional fluorescent microscopy. To visualize LYVE-1 and actin at higher resolution, we next sampled 10 × 10-μm areas of the HDLEC cell surface by STED microscopy. Intriguingly, the resulting images (Fig. 1*B*) revealed the submembranous actin cytoskeleton as a weblike network of branched fibers that enclosed corrals of varying size ranging from ∼0.1 to 1.5 μm and a separate heterogeneous distribution of LYVE-1 molecules within aggregates (or clusters) of ∼100 nm in diameter as reported in a previous independent study (30). Importantly, superimposition of such images indicated that the majority of LYVE-1 clusters were present within the boundaries of these corrals, whereas few if any appeared to be directly associated with their constituent actin fibers (*yellow arrows* in Fig. 1*B*). The lack of apparent overlap was further confirmed by quantitative image analyses, which yielded a low Pearson's correlation co-efficient of 0.1 ± 0.07, suggesting lateral confinement of LYVE-1 by the submembranous actin network rather than a physical interaction with actin filaments. It should be noted, however, that the actin mesh size in living HDLEC cells might be larger than observed in fixed cells, as small Arp2/3-mediated F-actin interactions can be lost in the process of fixation.

### Actin depolymerization triggers an increase in LYVE-1 HA-binding capacity

We next determined whether confinement by the cortical actin meshwork influences LYVE-1 receptor function, by comparing the HA-binding capacity of the endogenous native receptor on the luminal surface of primary untransfected HDLEC monolayers (where levels are known to be similar to those on the basolateral surface) (31) both before and after selective actin depolymerization. To simulate the dense multivalent configuration of HA within the DC surface glycocalyx (1, 5, 32), we prepared biotinylated HA-coated polystyrene beads conjugated with fluorescent streptavidin-Alexa 488 (hereafter referred to as bHA-coated beads, Fig. S1*A*). These were then incubated with monolayers of primary HDLECs together with CK-666 (50 μm) (33), cytochalasin D (Cyto D, 5 μm) (34), or latrunculin B (Lat B, 100 nm) (35), which respectively block Arp2/3-dependent filamentous actin branching, induce filamentous actin depolymerization by binding to the filaments' barbed ends, and sequester actin monomers and block their growth. In the case of each drug treatment, conditions were first optimized by time-lapse imaging (Fig. S1, *B–F*). As shown by the confocal z-stack projections and quantification in Fig. 1 (*C* and *D* (*top*)), treatment with Cyto D led to a 3-fold increase in the number of bHA-coated beads bound compared with DMSO-treated HDLEC controls, whereas treatment with Lat B or CK-666 increased binding 2-fold. Furthermore, bead binding was largely ablated in each case by inclusion of the LYVE-1 HA-blocking mAb 891 (6), confirming that the interaction was reversible and mediated primarily by LYVE-1 (Fig. 1 (*C* and *D*), *bottom panels*). This significant increase in binding was quantified as a measure of the number of beads bound to each actin-depleted condition (see “Experimental procedures”) (Fig. 1*D*).

Next, we investigated the effects of actin depolymerization on the capacity of native endogenous LYVE-1 in HDLECs to bind bHA in the form of fluorescent cross-linked biotin-streptavidin (bHA:SA488) complexes using quantitative flow cytometry. In a similar manner to bHA-coated beads, treatment with Cyto D (5 and 10 μm), Lat B (100 and 200 nm), and CK-666 (50 and 100 μm) also yielded a significant increase in bHA:SA488 complex binding (Fig. 1*E*). Moreover, the specificity of the binding interaction was confirmed by competition with a 200-fold molar excess of unlabeled high-molecular-weight HA (HMWHA), which also indicated that the bHA:SA488 complex was bound to the cell surface rather than internalized (Fig. 1*F*).

Curiously, closer inspection of the appropriate confocal images from Fig. 1*C* at higher magnification (shown in Fig. S1, *G* and *H*) revealed that LYVE-1 was concentrated beneath sites of bHA-coated bead attachment in actin-depolymerized cells and that such distribution was lost in the presence of LYVE-1 HA-blocking mAbs. These results suggested that disruption of the submembranous actin network facilitates ligand-induced clustering and avidity-dependent binding of LYVE-1 by higher-order HA complexes.

### The cortical actin network restricts LYVE-1 lateral mobility

**Figure 2.**
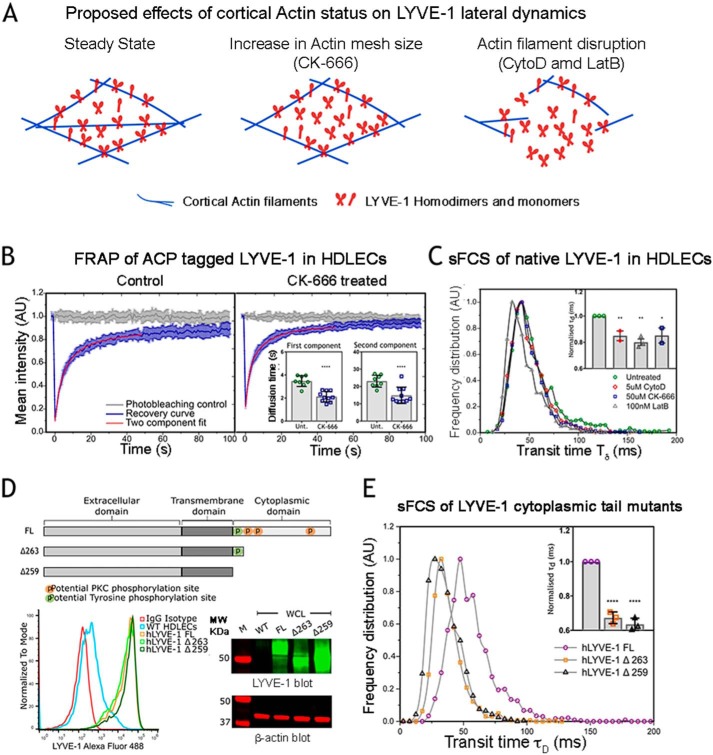
**Changes in lateral dynamics of LYVE-1 in HDLECs following actin disassembly.** *A*, scheme of proposed mechanisms that contribute to changes in lateral dynamics of LYVE-1 before and after actin depolymerization induced by treatment with the indicated agents. *B*, FRAP recovery curves for ACP-tagged hLYVE-1 labeled with Oregon Green 488 CoA in transfected HDLECs before (control) and after treatment with 50 μm CK-666, showing the fluorescence recovery (*blue*) of the diffusing molecules fitted to a two-component exponential model. *Insets*, average recovery times for the first and second LYVE-1 components as determined from the two-component free-diffusing fit of the FRAP curves for untreated cells (*Unt*.) and CK-666–treated cells, indicating faster mobility in both components after treatment (*n* = 10 cells, unpaired *t* test, *p* = <0.0001). *C*, sFCS histograms showing transit times of LYVE-1 in primary HDLECs labeled with Oregon Green 488–conjugated LYVE-1 8C Fab, in the presence or absence (control) of the actin-depolymerizing agents as indicated. *Inset*, calculated transit times shown as the mean ± S.E. (*error bars*) from three replicate experiments with *p* values (unpaired *t* test): Cyto D, 0.0122; Lat B, 0.0012; CK-666, 0.0407. *D*, schematics of full-length (*FL*) LYVE-1 and the LYVE-1 Δ263 and Δ259 truncation mutants depicting the extracellular domain, transmembrane domain, and cytoplasmic tail with its three potential protein kinase C (*PKC*) and single tyrosine phosphorylation sites. Expression levels of FL LYVE-1 and the LYVE-1 Δ263 and Δ259 mutants and endogenous LYVE-1 in transfected and control untransfected HDLECs as assessed by flow cytometry and Western blotting (all lanes are from the same unspliced gel, cropped to depict LYVE-1 monomer bands) are shown in the *bottom left* and *right panels*, respectively. *E*, sFCS histograms comparing transit times of FL LYVE-1 (*purple*) and the LYVE-1 Δ263 and Δ259 tail mutants labeled with Oregon Green 488–conjugated LYVE-1 8C Fab. The *bar chart* (*inset*) shows the normalized transit times as the mean ± S.E. from three replicates (*p* < 0.0001, unpaired *t* test).*, *p* ≤ 0.05; **, *p* ≤ 0.01; ****, *p* ≤ 0.0001.

The enhancement in LYVE-1 HA binding avidity upon actin filament disruption suggested that the lateral mobility of the receptor was physically confined by the cortical actin meshwork. To investigate this phenomenon more quantitatively, we determined the effect of actin depolymerization on LYVE-1 diffusion dynamics (Fig. 2*A*). To enable such measurements by fluorescence recovery after photobleaching (FRAP), we transfected HDLECs with recombinant N-terminal acyl carrier protein (ACP)-tagged LYVE-1, which was fluorescently labeled with the ACP ligand Oregon Green 488 CoA, before treatment with the previously implemented (see Fig. 1*D*) actin-debranching agent CK-666. The corresponding recovery curves (Fig. 2*B*) and their analyses (*inset*; see “Experimental procedures” for details) revealed a significant, 1.5-fold increase in LYVE-1 mobility upon drug treatment. Both curves exhibited two individual components, each of which showed an increase in diffusion, comprising a fast (52%, recovery times ∼2–3 ms, diffusion coefficients *D* = 0.12 μm^2^/s) and a slow component (48%, recovery times ∼15–20 ms, diffusion coefficients *D* = 0.02 μm^2^/s) (Table S1), possibly corresponding to LYVE-1 monomers and homodimers, respectively (4, 5).

To measure LYVE-1 mobility in primary HDLECs using a second independent method, we used sFCS, a single molecule–based technique in which receptor diffusion is monitored by confocal microscopy as the average time (τ*_d_*) taken for individual fluorescent receptor molecules to transit across a defined observation area, as determined simultaneously for each pixel along a scanned line. Importantly, as sFCS requires much lower receptor densities than FRAP, the method can thus be applied to the measurement of native LYVE-1 diffusion in primary untransfected HDLECs. sFCS data for endogenous LYVE-1 in confluent primary HDLECs detected with fluorescently conjugated mAb 8C Fab fragments were recorded and fitted as described under “Experimental procedures” (Fig. S2*A*). As shown in Fig. 2*C* and in agreement with the FRAP data, actin disruption by CK-666, Cyto D, or Lat B drug treatments in all cases led to a significant increase in LYVE-1 mobility (τ*_d_* = 50 ± 7 ms (untreated), 45 ± 5 ms (CK-666), 44 ± 3 ms (Cyto D), and 40 ± 5 ms (Lat B)) with a corresponding increase in the values of the diffusion coefficient *D* in the range 0.15–0.18 μm^2^/s as given in Table S2. (Note that in the case of sFCS, we only captured the fast diffusion component of the FRAP experiments, as slower-moving objects are frequently photobleached). Importantly, in our experiments, the mobility of LYVE-1 was independent of cell-culture duration as evidenced by the fact that LYVE-1 expression levels in HDLEC monolayers remained constant beyond 3 days in culture (Fig. S2, *B* and *C*) and were unaltered by the addition of 2% DMSO as required in the actin drug treatments (Fig. S2*D*). The trend toward an increase in LYVE-1 mobility after actin disruption was also independent of the method employed for measurement (FRAP *versus* sFCS) and receptor labeling (ACP *versus* Fab) despite the fact that they yielded minor differences in absolute values for diffusion coefficients (Fig. S2, *E–G*). These differences, which are inherent to such methodologies, have been highlighted previously (36) and were taken into consideration in our experiments by using the same measurement and labeling mode when comparing mobility between individual drug treatments.

### Truncating the LYVE-1 cytoplasmic tail leads to changes in lateral mobility

As highlighted by the previous measurements, disassembly of the cortical actin cytoskeleton led to a marked increase in LYVE-1 lateral mobility and hyaluronan-binding activity. Given that LYVE-1 possesses a 63-residue cytoplasmic tail and that the equivalent intracellular domain in the closely related leukocyte HA receptor CD44 mediates interactions with cortical actin (22), we explored the obvious possibility that similar interactions might regulate LYVE-1 lateral mobility. Accordingly, we generated two LYVE-1 truncation mutants, hLYVE-1 Δ263, which retains only the first 5 amino acids (VKRYV) of the cytoplasmic tail, and Δ259, which terminates immediately after the transmembrane region and is thus completely tailless, and transfected both separately into primary HDLECs for comparison with intact full-length hLYVE-1. Analysis by SDS-PAGE and flow cytometry confirmed that all three such constructs were expressed to similar levels and that each transfectant displayed equivalent densities of LYVE-1 on the HDLEC cell surface (Fig. 2*D*). Intriguingly, analysis of receptor mobility by sFCS (Fig. 2*E*) revealed that both LYVE-1 cytoplasmic tail mutants had significantly faster diffusion rates than the intact full-length receptor (τ*_d_* = 50 ± 4 ms (hLYVE-1 FL), 35 ± 3 ms (hLYVE-1 Δ263), and 33 ± 3 ms (hLYVE-1 Δ259) (*i.e. D* = 0.14 ± 0.01, 0.20 ± 0.02, and 0.22 ± 0.02 μm^2^/s, respectively); Table S3). These data provide the first evidence that the LYVE-1 cytoplasmic tail is indeed involved in regulating receptor lateral diffusion. However, they do not discriminate between direct physical adhesion or a more indirect confinement by the actin cytoskeleton.

### LYVE-1 displays little direct adhesion to the actin cytoskeleton

**Figure 3.**
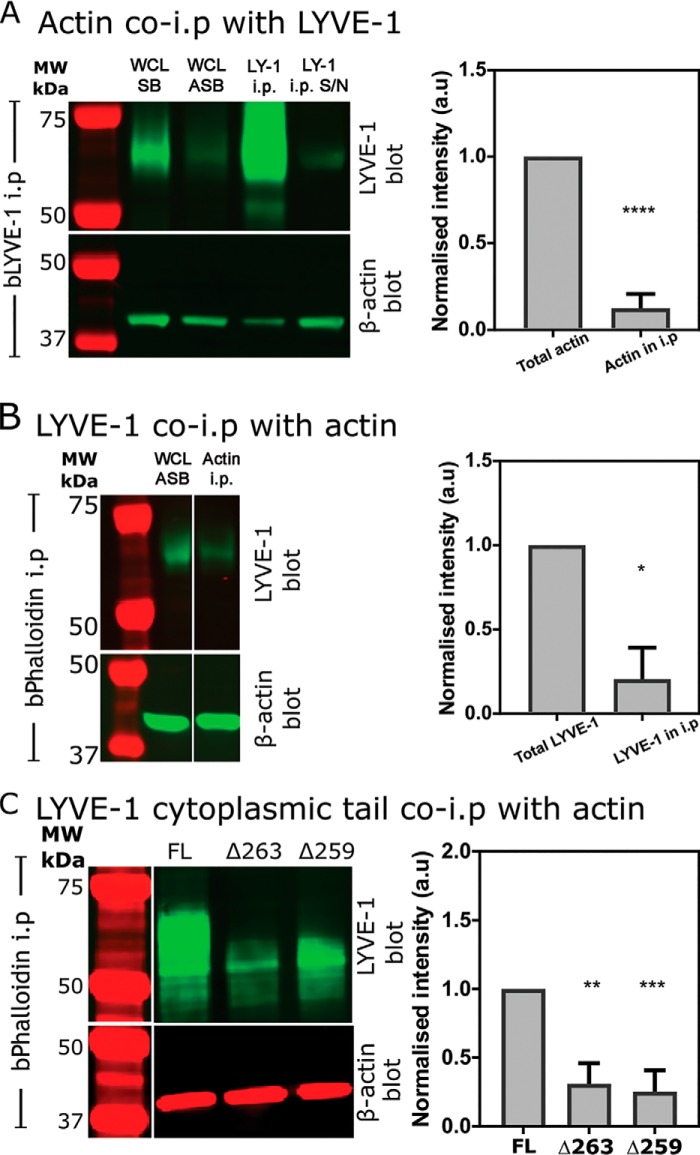
**Assessment of physical interaction between cortical actin and the LYVE-1 cytoplasmic tail in HDLECs by co-immunoprecipitation.** *A* and *B*, pulldowns of LYVE-1:actin complexes using either biotinylated LYVE-1 (*bLYVE-1*) (*A*) or bPhal (*B*) analyzed by SDS-PAGE and blotting with appropriate β-actin and LYVE-1 Abs, respectively (see “Experimental procedures”). Control immunoblots are also shown for matched loadings of HDLEC whole-cell lysates (*WCL*) dissolved directly in SDS-PAGE sample buffer (*SB*) and the soluble fraction from HDLECs lysed with the ASB used for subsequent immunoprecipitation (*i.p.*) (*S/N*, supernatant). Note that all three lanes are from the same corresponding gels in each case and spliced as shown. Bar charts show quantification using IR dye–conjugated secondary Abs and LI-COR® imaging (mean ± S.E. (*error bars*) (*n* = 5 in *A*; unpaired Student's *t* test, *p* = 0.0001; *n* = 3 in *B*, unpaired Student's *t* test, *p* = 0.0132). *C*, corresponding pulldowns of LYVE-1:actin complexes from HDLECs transfected with either FL LYVE-1 or LYVE-1 Δ263 or Δ259 cytoplasmic tail truncation mutants and LI-COR imaging (mean ± S.E., *n* = 4 for LYVE-1 Δ263, *p* = 0.0035, unpaired *t* test; mean ± S.E., *n* = 3 for LYVE-1 Δ259, *p* = 0.0002, unpaired *t* test). Note that all four lanes are from the same corresponding gels in each case and spliced as shown. *, *p* ≤ 0.05; **, *p* ≤ 0.01; ***, *p* ≤ 0.001; ****, *p* ≤ 0.0001.

To ascertain whether LYVE-1 interacts physically with the cortical actin cytoskeleton, we performed pulldown (co-immunoprecipitation) assays of the endogenous receptor using normal detergent-lysed primary HDLECs. Importantly, because the choice of detergent can be critical to maintaining the stability of such protein:protein interactions, we used a mixture of Triton X-100, SDS, and deoxycholate similar to that used in the original co-immunoprecipitation studies described for CD44 (37). In the first instance, LYVE-1 was immunoprecipitated using biotinylated LYVE-1 mAb and streptavidin Sepharose® beads, followed by SDS-PAGE and probing for β-actin using an appropriate mAb in the LICOR® near-IR detection system. The resulting blots (Fig. 3*A*) revealed only residual amounts of actin co-immunoprecipitate under these conditions. As a necessary control, corresponding pulldown assays were next performed in the reverse orientation to precipitate actin using biotinylated phalloidin (bPhal), and the SDS-polyacrylamide gels were subsequently blotted for LYVE-1. The results (Fig. 3*B*) again revealed only a residual degree of LYVE-1 co-precipitation, indicating little if any physical interaction between LYVE-1 and cortical actin in the HDLEC plasma membrane. Moreover, parallel pulldowns using Abs to the cytoskeletal linker ezrin failed to reveal co-immunoprecipitation of LYVE-1 (data not shown), arguing against the likelihood of an indirect interaction with actin via ERM proteins. Finally, these conclusions were further substantiated by pulldown assays of HDLECs supertransfected with the cytoplasmic tail truncation mutant hLYVE-1 Δ263 and the tailless mutant hLYVE-1 Δ259 (Fig. 3*C*).

### LYVE-1 lateral mobility is restricted by confinement within submembranous actin corrals

**Figure 4.**
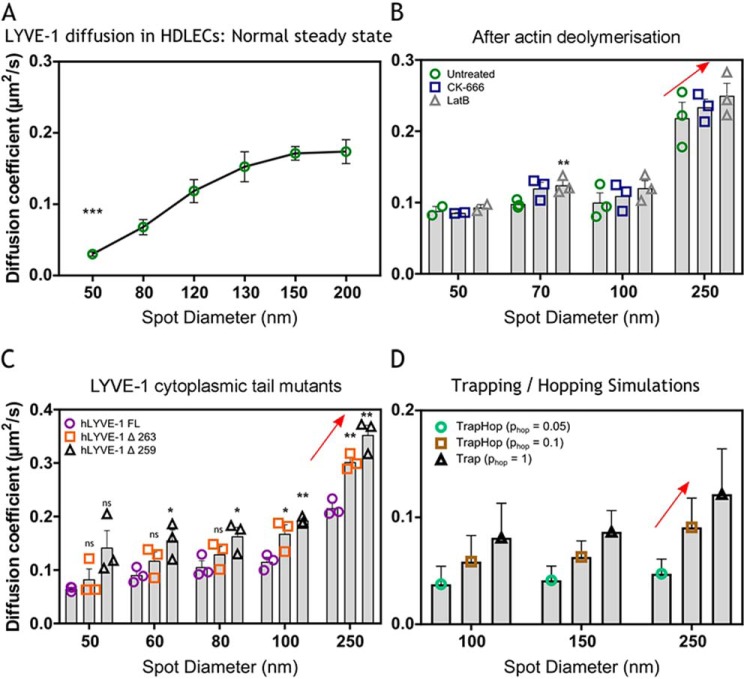
**STED-FCS analysis of LYVE-1 diffusion mode in the plasma membrane of HDLECs.** *A*, diffusion coefficients (*D*) for full-length LYVE-1 in primary HDLECs detected with Abberior STAR Red 8C Fab as determined at observation spot sizes in the range of 50–200 nm. *Green dots*, average from multiple cells at multiple locations, pooled from three experimental replicates, at 50 nm (*p* = 0.0006). *B*, apparent diffusion coefficients for full-length LYVE-1 in HDLECs detected with Abberior STAR Red 8C Fab as determined by STED-FCS after actin depolymerization by CK-666 or Lat B compared with untreated controls (mean ± S.E. (*error bars*), *n* = 3). Statistical *p* values (unpaired *t* test) for CK-666 at spot size 250 nm = 0.6262, 100 nm = 0.6206, 70 nm = 0.0818, 50 nm = 0.6772; *p* values for Lat B at spot size 250 nm = 0.3620, 100 nm = 0.3018, 70 nm = 0.0053, 50 nm = 0.6027. *C*, comparison of apparent diffusion coefficients from STED-FCS for full-length LYVE-1 and the cytoplasmic tail mutants Δ263 and Δ259 in transfected HDLECs detected with Abberior STAR Red 8C Fab (mean ± S.E., *n* = 3). Statistical *p* values (unpaired *t* test) for LYVE-1 Δ263 at spot size 250 nm = 0.0021, 100 nm = 0.0497, 80 nm = 0.2705, 60 nm = 0.2162, 50 nm = 0.4164; *p* values for LYVE-1 Δ259 at spot size 250 nm = 0.0023, 100 nm = 0.0012, 80 nm = 0.0483, 60 nm = 0.0369, 50 nm = 0.0732. *D*, comparison of diffusion coefficients from STED-FCS Monte Carlo simulations for the combination of trapping and hopping diffusion. The probability of hopping (*p*_hop_) indicates the ability of molecules to pass through the actin mesh boundaries, with a value of *p*_hop_ = 1 representing unrestricted passage. See “Experimental procedures” for details. *, *p* ≤ 0.05; **, *p* ≤ 0.01; ***, *p* ≤ 0.001; *ns*, not significant.

Our FRAP and sFCS dynamic studies and co-immunoprecipitation experiments provided evidence that LYVE-1 interacts with the actin cytoskeleton. In light of our evidence that LYVE-1 is not physically anchored to the cortical actin cytoskeleton, we therefore considered the possibility that the confinement in lateral mobility is exerted through more indirect interactions. In particular, we considered the possibility that restricted mobility results instead from transient entrapment of the receptor within the actin meshwork via its cytoplasmic tail, as proposed in the picket fence model (38–40, 68). The latter confinement has been shown to lead to a hoplike diffusion between such actin corrals and thus to an overall slowdown in diffusion (11). To help delineate such characteristics, we compared the diffusion dynamics of full-length LYVE-1 and its tail-deleted mutants in the plasma membrane of cultured HDLEC monolayers using FCS on a super-resolution STED microscope. These STED-FCS experiments uniquely identify diffusion modes such as hoplike diffusion by determining the values of the apparent diffusion coefficient (*D*) from FCS measurements at different observation spot sizes (*d*) between 50 and 250 nm in diameter. As highlighted before and detailed in Fig S2 (*H* and *I*), according to this analysis, a coordinate decrease in *D* with spot size *d* would indicate LYVE-1 hop diffusion across actin corrals, whereas pauses or temporary trapping of diffusion due to transient interactions with pointlike objects such as less-mobile proteins or protein aggregates (or clusters) would be highlighted by the inverse relationship (*i.e.* an increase in *D* with spot size *d*) (Fig S2, *H* and *I*). As shown by the results in Fig. 4*A*, there was, however, a clear correlation in the *D*(*d*) dependence for intact full-length LYVE-1, consistent with the transient trapping model, indicating transient and dynamic interactions between individual LYVE-1 molecules and less-mobile objects, such as the previously observed LYVE-1 aggregates or clusters (30). Such correlations and thus the transient interactions were largely unaffected when the actin cytoskeleton was disrupted by treatment with either CK-666 or Lat B (Fig. 4*B* and Table S4) or when the LYVE-1 cytoplasmic tail was either partially or fully deleted (Fig. 4*C* and Table S5), consistent with our repeated findings that LYVE-1 forms surface clusters independently of actin drug treatments (41). However, congruent with the confocal FRAP and sFCS data, the actin perturbation treatments led to marginal general increases in *D* values across all spatial scales (*i.e.* observation spot sizes *d*) compared with controls (*red arrows* in Fig. 4, *B–D*). To explore this behavior further, we performed Monte-Carlo computational analyses of hindered molecular membrane diffusion under conditions that simulated constant levels of transient LYVE-1 trapping and variable degrees of hop diffusion between compartments (*i.e.* mimicking actin corrals). Accordingly, we varied the probability *p*_hop_ of LYVE-1 intercompartmental hopping between the values of 1 (*i.e.* pure trapping and no hopping) and 0.05 (*i.e.* increased hop diffusion) while maintaining a constant value for compartment size (see “Experimental procedures” for details). The results of such simulations (Fig. 4*D*) revealed a preferential increase in *D* values across all spot sizes *d* due to the loss of hop diffusion, in a manner similar to that seen for the LYVE-1 cytoplasmic tail deletants and drug treatments. Hence, we conclude that the actin-dependent constraint in LYVE-1 mobility within the endothelial cell plasma membrane is due primarily to its transient confinement within actin corrals, in keeping with the so-called picket fence model of the plasma membrane (40). However, unlike the closely related receptor CD44, which acts as an anchored membrane picket through physical tethering of its cytoplasmic tail to the cortical actin meshwork (23), diffusion of LYVE-1 is restricted primarily through steric hindrance by the submembrane actin fence.

## Discussion

In this paper, we have presented new evidence that the lymphatic endothelial receptor LYVE-1 is functionally regulated by the submembranous actin cytoskeleton through constraints on receptor lateral mobility and membrane surface clustering. These findings have particular significance, as interactions between LYVE-1 and its ligand HA are known to be markedly dependent on avidity as a result of low affinity (*K_D_* = 8 μm for the native homodimeric receptor) and a consequent requirement for the glycosaminoglycan chains to engage high densities of LYVE-1 for efficient HA binding (4, 5), a phenomenon termed “superselectivity” (42). Indeed, LYVE-1 exhibits an exceptionally high degree of superselectivity *in vivo*, such that even very long HA polymers bind poorly to the receptor in lymphatic endothelial cells unless it is forced to cluster using bivalent mAbs, or the polymers themselves are assembled into large cross-linked supramolecular complexes, such as tumor necrosis factor-inducible gene 6 protein (TSG-6):HA or the streptococcal HA capsule, that can promote physiological LYVE-1 clustering (3–6). Accordingly, the capacity of dendritic cells and macrophages to adhere to and transmigrate the lymphatic endothelium depends on such LYVE-1 clustering, induced by selective engagement with HA in the form of the leukocyte surface glycocalyx (1, 2), an exoskeleton-like structure in which the glycosaminoglycan is complexed with CD44 and likely various other HA-binding partners that serve as cross-linking anchors (43–45).^4^ Our present findings suggest that the constraining effect of the actin cytoskeleton on lateral diffusion may impose this preference on LYVE-1 for binding large supramolecular HA configurations by setting a size threshold for efficient cross-bridging of the receptor into high-avidity clusters.

Using a combination of confocal and super-resolution STED microscopy to image native LYVE-1 in resting lymphatic endothelium at the single-molecule level, we observed that the receptor is organized in randomly distributed clusters rather than aligning with the submembranous actin network. Importantly, however, the vast majority of these LYVE-1 microclusters were trapped within the confines of discrete 0.1–1.5-μm-sized actin corrals rather than physically tethered to the actin fibers. Furthermore, we showed that disruption of the cortical actin network by treatment with either Lat B, Cyto D, or CK-666 led not only to an increase in the mobility of LYVE-1, as determined by FRAP and sFCS analysis, but also to a marked increase in its capacity to bind to HA-coated microbeads and HA streptavidin complexes, indicating that in steady state, the ability of LYVE-1 to form clusters and harness multivalent HA binding is indeed limited by its association with the actin network. Importantly, the increase in lateral diffusion elicited by actin disruption was observed only for intact full-length LYVE-1 and not for truncation mutants lacking the cytoplasmic tail, indicating for the first time a significant functional role for this intracellular domain. However, the results of quantitative pulldown assays using either LYVE-1 mAbs or phalloidin demonstrated that only a minor proportion of the receptor segregated with the actin network, more consistent with intercalation of the cytoplasmic tail within actin corrals rather than direct physical anchoring.

The restricted plasma membrane mobility we describe here for LYVE-1 has also been well-documented for the structurally related receptor CD44, which mediates similar avidity-dependent binding to HA, but in leukocytes, fibroblasts, epithelial cells, and blood vessel endothelium rather than in lymphatic endothelium (46–48). However, in contrast to LYVE-1, CD44 binds robustly to the actin cytoskeleton via its 63-residue cytoplasmic tail, which contains specific binding motifs for the actin adapter proteins ezrin, radixin, and moesin and the spectrin adapter protein ankyrin (20–22, 49–52). Moreover, such interactions with actin have been shown to mediate clustering of CD44 and regulation of binding to its high-molecular-weight HA and E-selectin ligands in peripheral blood mononuclear cells and various transformed cell lines (22, 48, 53). Indeed, in primary macrophages, anchorage to the actin cytoskeleton was shown to permit mobilization of CD44 to the trailing edge of the cells during their polarization in response to chemokine-induced motility (23). This property is itself critical for the ability of dendritic cells to engage with lymphatic endothelium, as besides anchoring HA in the surface glycocalyx, CD44 serves also to cluster the glycosaminoglycan for avidity-dependent binding to LYVE-1,^4^ most likely by allowing it to cross-bridge multiple receptors segregated within remote actin corrals.

**Figure 5.**
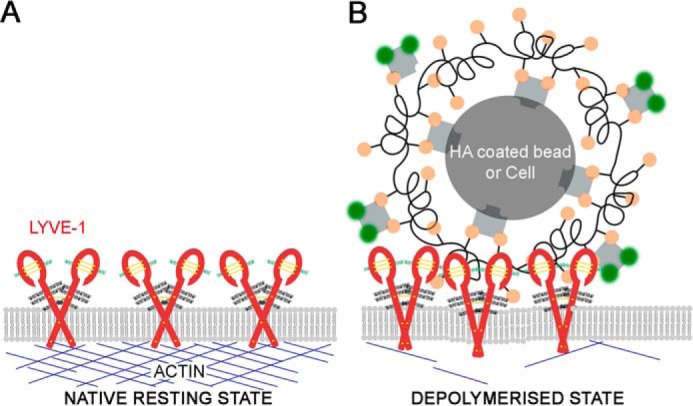
**Proposed model of the relationship between cortical actin, LYVE-1 dynamics, and HA binding in the lymphatic endothelial cell plasma membrane.** Changes in the cytoskeletal actin from its native polymerized state (*A*) to a depolymerized state (*B*) within LECs increase the lateral mobility of LYVE-1, enabling multimolecular HA complexes such as those in the surface glycocalyx of lymph-migrating leukocytes to induce higher-order clustering of the receptor and harness the binding avidity required for pro-adhesive interactions (*B*).

The firm anchorage of high-copy-number receptors, such as CD44, to the cortical actin cytoskeleton has given rise to the so-called picket fence model in which dense subcompartments of such molecules in the plasma membrane present physical barriers to the diffusion paths of other more mobile surface components by virtue of their bulky ectodomains (38). However, this scenario does not fit with the behavior we observed here for LYVE-1. Instead, we postulate an alternative model (Fig. 5) in which loose entrapment of the receptor within actin corrals rather than tight anchoring limits its long-range lateral mobility (Fig. 5*A*), thereby poising LYVE-1 for a rapid increase in diffusion and avidity-dependent HA binding upon actin disassembly (Fig. 5*B*), in line with its known superselective properties (42).

The precise physiological signals for *in vivo* dynamic actin assembly/disassembly in lymphatic endothelium are not currently known. Nevertheless, in lymphatic endothelium *in vitro*, the submembranous actin network adjacent to button junctions has been reported to undergo local disassembly and redistribution in response to a transient rise in intracellular free Ca^2+^ triggered by physical contact with migrating DCs (39). One might therefore envisage that the resulting increase in LYVE-1 lateral mobility would facilitate higher-order clustering of the receptor following engagement with the DC HA glycocalyx and coalescence to form the 2–3-μm transmigratory cup structures that mediate their adhesion to endothelium and subsequent transit in the so-called lymphatic synapse (54). Further studies using real-time imaging of LYVE-1 and actin filament dynamics following DC engagement with LECs will be required to resolve this issue.

In conclusion, our results provide the first evidence for regulation of LYVE-1 receptor function by the endothelial actin cytoskeleton and help explain the ability of LYVE-1 to discriminate HA glycocalyx-coated leukocytes from free HA. Furthermore, they raise the possibility that dynamic control of actin assembly/disassembly at endothelial junctions, where interactions between LYVE-1 and HA are critical for adhesion and transit, might be part of the mechanism by which HDLECs regulate this vital process and thus access to the lymphatic system.

## Experimental procedures

### Cells

Experiments were performed using confluent monolayers of primary HDLECs isolated from human skin, obtained from healthy individuals undergoing plastic surgery at the John Radcliffe Hospital (Oxford, UK). The cells were isolated and characterized as described (40) and cultured in EGM-2 medium on 0.1% (w/v) gelatin (#1393, 100 ml, Sigma–Aldrich)-coated flasks at 37 °C in the presence of 5% CO_2_.

### Generation of LYVE-1 constructs for cell surface expression

ACP-tagged primers were used to amplify the sequence encoding the mature LYVE-1 polypeptide (minus signal peptide) from cDNA. Endonuclease restriction sites are underlined, and the positions of stop codons, where appropriate, are shown in boldface italic type. Primers for the ACP-tagged LYVE-1 fusion construct were hLY 130F BamHI (5′-GCGGGATCCTCTTTGCGTGCAGAAGAGC) and hLY 969R NotI (5′-GCGGCGGCCGC***CTA***AACTTCAGCTTCCAGGCATCGC). This was ligated into a BamHI/NotI-cut pACP-tag(m)-2 vector to generate an in-frame fusion with the ACP tag (#N9322, New England Biolabs). This new construct was digested with EcoRI and NotI to yield an insert for the fusion of ACP and LYVE-1 that was then ligated into the pACP ADR β2 plasmid (supplied as a transfection control) to add the signal peptide (from the 5HT3A serotonin receptor) to the N terminus. The entire assembly was then amplified with the primers pACP ADRβ2 F BglII (5′-GCGAGATCTGGATCGTCGCTAGCACCATGC) and hLY 969R NotI, as above. The amplified product of both was cloned into a derivative of vector pHR Sin (55), carrying the gene for ACP cut with BamHI (compatible with BglII) and NotI, to generate a fusion protein with the N terminus of LYVE-1. These were then lentivirally transduced into primary HDLECs as described previously for stable expression (4, 6).

Enhanced GFP (eGFP) and Haloalkane dehydrogenase (Halo) tagged full length LYVE-1 constructs were amplified using the forward primer, hLY 14F MluI and the reverse primer: hLY FL R BamHI, 5′-GCGGGATCCACTTCAGCTTCCAGGCATCG. These were then digested and ligated into derivatives of the pHR Sin vector carrying the coding sequence for eGFP or Halo, and used for lentiviral transduction of primary HDLEC as described above.

LYVE-1 cytoplasmic tail truncation mutants were generated with the common forward primer hLY 14F MluI, 5′-GCGACGCGTGAAGGGGTAGGCACGATGGCCAGG and the following reverse primers: hLYVE-1 Δ259 NotI* R, 5′-GCGCGGCCGC***TTA***GACATAGCAAAATCCAAGACCAGC; hLYVE-1 Δ263 NotI* R, 5′-GCGCGGCCGC***TTA***CACATACCTTTTGACATAGCAAAATCC; and hLY 969R NotI as above. The amplified products were cloned into a derivative of vector pHR Sin lacking the gene ACP. Constructs were then used for lentiviral transduction of primary HDLEC as described previously (6).

### Generation of fluorescent LYVE-1 antibody Fab fragments

Hybridomas expressing monoclonal mouse anti-human LYVE-1 mAb 8C were grown for 6 weeks in culture, and mAbs were affinity-purified as described previously (56, 57). LYVE-1 Fab fragments were generated as per the manufacturer's instructions (mouse IgG1 Fab preparation kit (#44980, Thermo Scientific)). The resulting LYVE-1 Fabs were then purified by size-exclusion chromatography and conjugated with Oregon Green® 488-X succinimidyl ester and Abberior® STAR Red NHS ester at a 1:3 antibody/molar dye ratio in the presence of 0.1 m NaHCO_3_ (pH 8.3). Unconjugated dye was then removed using size-exclusion chromatography, and the degree of conjugation was determined as 1 dye per Fab molecule (58).

### Labeling of LYVE-1 and actin in HDLECs for microscopic imaging

Monolayers of primary HDLECs grown on 0.1% gelatin-coated glass slides (Ibidi μ-Slide 8-well glass bottom (#80827, Ibidi) were rinsed with PBS and fixed in 1% (w/v) paraformaldehyde for 15 min at room temperature (RT). Cells were then rinsed with excess PBS and permeabilized for 1 min in wash buffer (PBS, 10% fetal calf serum, and 0.5% sodium azide) containing 0.1% Triton X-100, prior to the addition of Abberior® STAR 635–labeled phalloidin (#30972-20UG, Sigma–Aldrich, 1:100 final dilution) and mouse anti-human LYVE-1 (mAb 8C, 10 μg/ml) and incubation for 15 min at RT. After rinsing with PBS, a secondary Alexa Fluor 594 goat anti-mouse IgG (#A11005, Thermo Scientific) was added to detect LYVE-1. The cells were then rinsed with PBS and placed in Leibovitz's (L-15) phenol red–free medium (#21083027, Thermo Scientific) for confocal and STED imaging. The images acquired were viewed in Fiji/ImageJ (RSB, National Institutes of Health) (59), and the CoLoc2 plugin was used to determine the Pearson's correlation between actin and LYVE-1. For labeling actin alone (Fig. S1*F*), phalloidin Oregon Green at 1:100 dilution was used as above.

### Binding of bHA-coated beads

Streptavidin (SA) polystyrene beads (#PC-S-6.0, Kisker Biotech GmbH & Co. KG) were coated with bHA in EGM-2 medium with constant rotation for 10 min at RT. After removal of unbound bHA by washing in EGM-2, the beads were subsequently labeled with Streptavidin Alexa Fluor 488 (SA488:bHA:SAbeads). Labeled beads (∼1 × 10^6^) were then added to HDLEC monolayers together with the actin depolymerization agents Cyto D (5 μm) (#C8273, Sigma–Aldrich) or Lat B (100 nm) (#L5288, Sigma–Aldrich), CK-666 (50 μm) (#182515, Merck Millipore), or LYVE-1 HA-blocking antibody (#MAB20891, R&D Systems), as appropriate, and incubated for 15 min at 37 °C in the presence of 5% CO_2_. After washing and re-incubation (5 min) in wash buffer, any loosely bound beads were removed by rinsing several times with PBS, and cells were fixed with 1% (w/v) paraformaldehyde and 0.2% (w/v) glutaraldehyde for 10 min. Polyclonal goat anti- human LYVE-1 Ab (#AF2089, R&D Systems, 10 μg/ml) was next added to the cells and allowed to incubate for 20 min at RT, followed by detection with 1:200 dilution of donkey anti-goat Alexa Fluor 568 (Thermo Fisher Scientific), along with NucBlue^TM^ Live ReadyProbes^TM^ reagent (#R37605, Thermo Fisher Scientific) for 10 min at RT. Monolayers were then rinsed in wash buffer, and L-15 medium was added in preparation for microscopy, whereby 10–15 images were recorded per condition in each experimental replicate. The levels of bHA bead binding were quantified using a custom script written in Python (based on RRID:SCR_008394). Images were converted to binary for thresholding, followed by application of a Gaussian filter. The regional maxima were identified and used for watershed segmentation, after which the number of beads per image were counted.

### Binding of bHA:streptavidin complexes

Monolayers of confluent primary HDLECs were treated with either Cyto D, Lat B, or CK-666 and incubated for 15 min at 37 °C. The cells were immediately rinsed and re-incubated in wash buffer for 5 min, followed by final rinsing in PBS and detachment with Accutase®. Biotinylated HA (5 μg/ml, 1:1 conjugation ratio) and streptavidin Alexa Fluor 488 (#S1123, Thermo Fisher Scientific) were then added, and the cells were incubated overnight at 4 °C with shaking (800 rpm). Unbound complexes were removed by rinsing in wash buffer, and cells were subsequently fixed in FACS fix (2% formaldehyde and 0.02% sodium azide) prior to quantitation of bHA binding by flow cytometry using either Cyan (BD Biosciences) or Attune (Thermo Fisher Scientific) FACS analyzers, and the data were analyzed using FlowJo version 10 (FlowJo, LLC).

### Imaging of cortical actin networks

Monolayers of primary HDLECs were grown on 0.1% gelatin-coated glass-bottomed WillCo dishes and labeled with SiR actin (#CY-SC001, Cytoskeleton, Inc.) in EGM-2 medium for 1 h at 37 °C. These were then rinsed and re-incubated in warm L-15 medium either alone or in the presence of Cyto D or Lat B as appropriate. Images of the monolayers were acquired every minute and analyzed using ImageJ. Small rectangles (*yellow*) were drawn in several F-actin–rich areas, and the average intensity of actin was measured for every minute recorded. The average of the intensities from every recorded rectangle for a single time point was calculated and plotted against time.

### Co-immunoprecipitation analyses

Confluent primary HDLEC monolayers that had been cultured for 7–10 days were washed twice in ice-cold PBS, pH 7.5, prior to lysis (20 min) in a modified actin stabilization buffer (ASB) (37, 60) comprising 50 mm Tris-HCl, pH 7.5, 0.5% Triton X-100 (#93443, 100 ml, Sigma–Aldrich), 0.1% SDS (#BP1311-1, Fisher Scientific), 0.5% sodium deoxycholate (#D6750, Sigma–Aldrich), 10 mm EDTA (#5134, Sigma–Aldrich), 150 mm NaCl, freshly supplemented with 0.5 mm ATP (#A3377, Sigma–Aldrich), 200 μm sodium orthovanadate (#S6508, Sigma–Aldrich), 200 μm sodium fluoride (#S6776, Sigma–Aldrich), and protease inhibitors (#11873580001, Roche Applied Science). After centrifugation (15 min at 15,000 × *g*), lysates were decanted and incubated with either 2.5 μg of bPhal (#B7474, Molecular Probes) or biotinylated goat anti-human LYVE-1 Abs (b-LYVE-1, #BAF2089, R&D Systems) at 4 °C overnight followed by the addition of streptavidin Sepharose beads (#20359, Sigma) for a further 4 h with end-over-end rotation. Following initial centrifugation (4,000 × *g*, 15 min), the beads were then subjected to five successive rounds of washing with cold ASB before elution with SDS-PAGE sample buffer (95 °C, 5 min) and electrophoresis on BisTris 4–12% polyacrylamide gels (#NP0322BOX, LifeTech). For blotting, gels were then transferred onto polyvinylidene difluoride membranes (#IPFL00010, Immobilon-FL Transfer membrane, Millipore) and probed for either LYVE-1 (LYVE-1 Ab #AF2089, R&D Systems) or actin (β-actin mAb A5441-100UL) using IRdye® 800 conjugates (#P/N 925-32214, #P/N 925-32212, LI-COR Biotechnology) prior to quantitative imaging with a LI-COR Odyssey scanner and Image Studio software.

### Confocal and STED microscopy

Confocal imaging was performed on a Zeiss 880 LSM inverted confocal microscope equipped with a Plan Apochromat ×63 oil immersion lens. Time-lapse and super-resolution STED images were acquired using the Leica SP8 TCP inverted microscope (Leica Microsystems, GmbH, Mannheim, Germany) equipped with an HCX PL APO ×100 oil immersion lens (numerical aperture 1.4) and illuminated with a white-light laser (NKT Photonics) for flexible choice of excitation wavelengths and a high-power pulsed (CW) STED laser. Dual-color sample excitation was carried out at 561 and 633 nm at 30 μW in a frame-by-frame sequence and depleted using the 775 STED laser at 150 milliwatts. Single-color excitation was carried out with 633 nm at 30 μW and depleted as above. For every treatment, at least 10 images were acquired per repeat. For fixed cells, the measurements were performed at RT, whereas all live-cell imaging was performed at 37 °C in the presence of 5% CO_2_.

### sFCS

The confocal sFCS measurements were taken on a Zeiss 780 LSM inverted confocal microscope equipped with a ×40 C-Apochromat numerical aperture 1.2 W Corr FCS water objective (Zeiss) using a laser power of 5 μW. 10 nm Alexa Fluor 488 in water was added to a 0.1% (w/v) gelatin-coated 18-mm coverslip (no. 1.5 thickness) and used for calculating the observation volume using a three-dimensional diffusion model. The diffusion coefficient (*D*) of LYVE-1 was obtained by fitting the correlation curve with the two-dimensional diffusion fit in FoCus point software (61).

Primary HDLECs were grown to a monolayer on 0.1% gelatin-coated 18-mm coverslips (#MIC3342, Scientific Laboratory Supplies Ltd.). The cells were labeled with 20 μg/ml LYVE-1 Fab Oregon Green 488 at 37 °C for 10 min with 5% CO_2_. Excess antibody was removed by washing and replacement with L-15 medium. The labeled cells were allowed to equilibrate at the microscope for a few minutes prior to the addition of Cyto D, Lat B, or CK-666 for 10 min. A line scan of 5.2 μm with a pixel dwell time of 3.94 μs and scan time of 472.73 μs was carried out for each measurement. Around 10–14 cells were measured per experiment. All measurements were carried out at 37 °C in the presence of 5% CO_2_.

### Scanning FCS analysis and fitting

The sFCS data were correlated and processed using the FoCuS-scan software version 13 (62). Initial bleaching was removed by cropping off the first 5–10 s of all measurements, and bleaching was further corrected by applying a local averaging of 16-s intervals. The curves obtained from this were fitted using a single-component two-dimensional diffusion model as described (63) as follows,

(Eq. 1) $$G\left(\tau \right)=\frac{1}{N}\cdot \frac{1}{\left(1+\tau /\tau_{D}\right)}+O_{f}$$

where τ denotes the correlation time, offset *O_f_* was left to vary, and amplitude (given as inverse average number of particles in the observation volume) 1/*N* and transit time τ*_d_* were the fitting parameters. The fitting was performed in the range of 0.5–4,000 ms, and the data generated were then run through a MATLAB (MathWorks) script and analyzed on the basis of the finding that histograms of sFCS transit times follow a log-normal distribution (37, 63). The log-normal function exists in three analytical forms: linear, cumulative, and logarithmic representations. The statistical analysis exploits these three representations for accurate fitting (Fig. S2*A*). From these fitted values, a determined median transit time was obtained from the sFCS data. The diffusion coefficient was then calculated, and replicate values were plotted in GraphPad Prism to determine statistical significance.

### STED-FCS

STED-FCS measurements were carried out on primary HDLECs labeled with LYVE-1 Fab Abberior STAR Red as above on the Leica SP8 TCP inverted microscope. The master power of the white-light laser was set to 30%. Sample excitation at 633 nm was used at 1–25% (1–25 μW) output power. STED depletion was carried with a pulsed 80-MHz IR laser (755 nm) at laser powers (0–180 milliwatts). STED-FCS measurements were collected onto a single-photon-counting avalanche photodiode (APD; Micro Photon Devices, PicoQuant, Berlin, Germany) in the external port of the microscope. The APD signal was recorded with a time-correlated single-photon-counting (TCSPC) detection unit (Picoharp 300, PicoQuant), which saves the raw photon stream. The TCSPC control software (SymPhoTime, PicoQuant) allows reconstruction of fluorescence lifetime decays as well as fast calculation of FCS data. The recordings were directly controlled by the Leica LAS AF software, which communicates with the PicoQuant SymPhoTime software as an already integrated FCS package in LAS AF.

### Determination of observation spot diameter for STED-FCS

Calibration with supported lipid bilayer (SLB) was as follows. SLB was prepared as described previously (64–66). A lipid mix of 1 mg/ml 1,2-dioleoyl-*sn*-glycero-3-phosphocholine (Avanti Polar Lipids) and 1,2-dipalmitoyl-*sn*-glycero-3-phosphoethanolamine (DPPE) (Abberior STAR Red, Abberior GmbH) at a ratio of 2000:1 was dissolved in chloroform/methanol mix (a ratio of 2:1 (v/v) (Sigma–Aldrich)). The mix was vortexed vigorously and spin-coated (KW-4A Spin-Coater, Chemat Technology) onto a coverglass (25-mm diameter, no. 1.5 thickness, #MIC3350, SLS Ltd.) precleaned with Piranha acid (3:1 sulfuric acid and hydrogen peroxide, 15-min incubation). The cover-glass was mounted onto a chamber and rehydrated with SLB buffer (10 mm HEPES (Lonza Biologics plc, Slough, UK) and 150 mm NaCl, pH 7.4).

The diameter (*d*) of the STED observation spot was tuned by the STED laser power (*P*_STED_). STED-FCS measurements on the above DPPE- Abberior STAR Red SLB at different *P*_STED_ values were acquired to accurately calibrate the *d*(*P*_STED_) dependence. The STED spot diameter *d* was determined using the following equation,

(Eq. 2) $$d=FWHM_{STED}=FWHM_{CONF\cdot }\sqrt{\tau_{d\left(STED\right)}/\tau_{d\left(CONF\right)}}$$

where τ*_d_*_(CONF)_ and τ*_d_*_(STED)_ are the transit times of the fluorescent molecules through the observation spot in confocal and at varying STED laser power (0–180 milliwatts), respectively. The confocal observation spot (FWHM_CONF_) was set to a diameter *d* = 250 nm according to confocal images of fluorescent beads. The FCS measurements on the SLBs were carried out for 5 s in triplicates at different spots.

The STED-FCS measurements were carried out in at least 7–10 different cells per experiment and were measured at different regions for every cell at the different STED powers. The apparent diffusion coefficient (*D*) for each diameter was calculated according to the equation,

(Eq. 3) D=d^2/(8·ln⁡2τd)

where *d* represents the diameter of the observation spot, and τ*_d_* is the transit times at these varying diameters. This is used to plot the *D*(*d*) dependence curve.

### FRAP

ACP FL hLYVE-1–transfected HDLECs were plated onto 0.1% (w/v) gelatin-coated glass-bottom WillCo dishes (#GWSB-3522, WillcoWells, Amsterdam, Netherlands) and allowed to grow to confluence. The confluent cells were labeled by the addition of 1 mm Oregon Green 488–labeled CoA (#S9348, New England Biolabs), 1 mm MgCl_2_, and ACP synthase (#P9301S, New England Biolabs) and allowed to incubate at 37 °C with 5% CO_2_ for 60 min. The cells were then washed in PBS and imaged in L-15 medium.

FRAP data acquisition was designed following a procedure described previously (67) and as described (30). FRAP experiments were performed on a Zeiss 780 scanning confocal inverted microscope using a Plan Apochromat ×63 oil immersion lens. A circular imaging region of 1.5-μm radius comprising a smaller circular region of interest with a radius of 1 μm within the imaging region at the apical cell membrane was applied for acquiring FRAP time lapses. The FRAP protocol consisted of three steps: two frames of acquisition, a 2-s photobleaching event, and subsequent FRAP recovery recordings for 100 s at a rate of 1 frame/s. FRAP images were acquired using the 488-nm laser at 1.5% power. 100% of 488-nm laser power was used to bleach the region of interest after two frames were acquired. The photobleaching control measurements were performed with the same settings but without the photobleaching irradiation.

(Eq. 4) $$F\left(t\right)=A_{1}\times \left(1-\exp\left(-\left(1-t_{0}\right)/\tau_{1}\right)\right)+A_{2}\times \left(1-\exp\left(-\left(1-t_{0}\right)/\tau_{2}\right)\right)$$

The FRAP curves were processed using the image analysis software Fiji/ImageJ, following analysis strategies as described (67). FRAP raw data extracted from each FRAP recovery curve were plotted using OriginPro 9 (Origin Labs). All curves were time-aligned, normalized, plotted, and fitted using the OriginLab fitting function obtained from Ref. 67. FRAP curves were corrected for fluorescence background using Fiji/ImageJ background subtraction. FRAP recovery curves were fitted with a double-exponential fitting function below as described (25).

### Simulation of STED-FCS data

We generated simulated fluorescence time traces and correlation curves from point STED-FCS data as Monte Carlo simulations similarly as described (37, 66). Briefly, we used the nanosimpy repository, which is freely available on GitHub https://github.com/dwaithe/nanosimpy,^5^ to simulate particle movement with a combination of trapping and hopping diffusion modes in Python. 200 molecules were randomly initialized in a circular region with a diameter of 3,000 nm and wrapped around once they left the region. Particle movement was simulated for 15 s with time steps of 0.001 ms. A confinement mesh map was simulated by Voronoi transform of randomly seeded points of 50-nm average distance, resulting in a mesh size of 110 nm given as √*area*. For every time step, each molecule was moved with a diffusion coefficient of 0.4 μm^2^/s and had a chance of trapping (molecular complex formation, diffusion coefficient changes to 0.1 × 10^−9^ μm^2^/s being practically immobile) with a probability of *p*_trap_ of 0.00005. Once a particle was trapped, it had the same probability to diffuse freely again. *p*_trap_ was constant throughout the simulations. Once a particle hit a mesh boundary, it had a probability of *p*_hop_ to pass through; otherwise, it would remain in the same compartment. *p*_hop_ was varied from 0.01 to 1 (pure trapping) to mimic the effect of the LYVE-1 cytoplasmic tail truncation mutants. After generation of all molecular tracks, (STED)-FCS measurements were performed in the center of the simulation area. The molecules were passed through a Gaussian-shaped observation spot with full-width half-maxima of 250, 150, 100, and 50 nm, mimicking the experimental point spread functions. The resulting intensity traces were correlated and fitted as the experimental data. Every simulation was performed in 10 repetitions.

### Statistical analysis

GraphPad Prism 7.0 (GraphPad Software, Inc.) was used to perform statistics. The normalized mean intensity values of the bead-binding assay and FACS were plotted in GraphPad, and statistical significance was obtained using unpaired *t* test and analysis of variance, respectively. A follow-up Dunnett's multiple-comparison test was also performed to test significance between drug treatments in bHA:SA488 complex-binding FACS assays. The transit times and diffusion coefficients obtained from individual FRAP curves and replicates from sFCS and STED-FCS were also plotted in GraphPad, and an unpaired Student's *t* test analysis was performed to obtain statistical significance. An unpaired *t* test was also performed for comparing significance in the co-immunoprecipitation experiments. The numbers of experimental replicates and *p* values for each experiment are listed in the figure legends.

## Author contributions

T. A. S., M. F., C. E., and D. G. J. conceptualization; T. A. S. data curation; T. A. S. formal analysis; T. A. S. investigation; T. A. S., S. B., D. S., and F. S. methodology; T. A. S. writing-original draft; T. A. S., C. E., and D. G. J. writing-review and editing; M. F., S. B., C. E., and D. G. J. supervision; M. F., C. E., and D. G. J. project administration; F. S. software; C. E. funding acquisition.

## Supplementary Material

Supporting Information

## Footnotes

4 L. A. Johnson and D. G. Jackson, unpublished observations.

5 Please note that the JBC is not responsible for the long-term archiving and maintenance of this site or any other third party hosted site.

## Footnotes

3 The abbreviations used are:

LYVE-1: lymphatic vessel endothelial hyaluronan receptor-1
ACP: acyl carrier protein
hLYVE-1: human LYVE-1
ASB: actin-stabilizing buffer
Lat B: latrunculin B
LECs: lymphatic endothelial cells
bHA: biotinylated HA
bPhal: biotinylated phalloidin
Cyto D: cytochalasin D
DC: dendritic cell
DPPE: dipalmitoylphosphatidylethanolamine
ERM: ezrin, radixin, and moesin
RT: room temperature
F-actin: filamentous actin
S/N: supernatant
FCS: fluorescence correlation spectroscopy
FRAP: fluorescence recovery after photobleaching
sFCS: scanning FCS
FWHM: full-width at half-maximum
SLB: supported lipid bilayer
STED: stimulated emission depletion
HA: hyaluronan
HDLEC: human dermal lymphatic endothelial cell
HMWHA: high-molecular-weight HA
SA: streptavidin
μW: microwatts
APD: avalanche photodiode
TCSPC: time-correlated single-photon-counting
FL: full-length.
