# Supplementary material for: The cortical actin network regulates avidity-dependent binding of hyaluronan by the lymphatic vessel endothelial receptor LYVE-1
Source: J Biol Chem. 2020 Feb 7;295(15):5036–50. doi: 10.1074/jbc.RA119.011992 (PMC7152780; doi:10.1074/jbc.RA119.011992)
Supplement: Supporting Information [file supp_295_15_5036__index.html]

The cortical actin network regulates avidity-dependent binding of hyaluronan by the Lymphatic Vessel Endothelial receptor LYVE-1 — Actin cytoskeleton regulates LYVE-1:HA binding — The cortical actin network regulates avidity-dependent binding of hyaluronan by the lymphatic vessel endothelial receptor LYVE-1 — Actin cytoskeleton regulates LYVE-1:HA binding — Supporting Information 

# The cortical actin network regulates avidity-dependent binding of hyaluronan by the lymphatic vessel endothelial receptor LYVE-1

## Supporting Information

- Supporting Information - Combined Figure legends and Figures S1 and S2 of Supporting information section
