# Supplementary material for: The cortical actin network regulates avidity-dependent binding of hyaluronan by the lymphatic vessel endothelial receptor LYVE-1
Source: J Biol Chem. 2020 Feb 7;295(15):5036–50. doi: 10.1074/jbc.RA119.011992 (PMC7152780; doi:10.1074/jbc.RA119.011992)
Supplement: Supporting Information [file supp_RA119.011992_157240_2_supp_471742_q5bzmw.pdf]

Supporting Information.

Fig. S1: Actin depolymerisation dynamics and their influence on LYVE-1:bHA coated bead binding

A) Diagrammatic representation of experimental system used to model the HA glycocalyx-mediated binding of DCs to LYVE-1 in HDLECs using fluorescent bHA:Streptavidin (SA488) coated polystyrene beads. B - F) Effects of Lat B, Cyto D and CK-666 on F-actin depolymerisation. B, C) Confocal time-lapse images of F-actin in primary HDLECs labelled with SiR Actin before (above diagonal) and after (below diagonal) treatment with 100 nM Lat B (B) or 5 µM Cyto D (C), scale bars in each case 10 µm. D, E) Average fluorescence intensity of F-actin over time from 5 ROI (yellow boxes in B and C respectively) plotted against acquisition time and fitted with an exponential decay fit for both treatments. F) Confocal z-stack projections of actin in primary HDLECs stained with phalloidin Oregon Green, confirming the actin cytoskeleton is depolymerised upon treatment with Cyto D, Lat B or CK-666 compared to untreated controls, scale bars in each case 50 µm. G, H) Magnified versions of selected images from Figure 1C showing distribution of LYVE-1 in HDLECs in the contact region beneath bound bHA coated beads before and after actin depolymerisation with latrunculin B, in the presence or absence of LYVE-1 blocking antibody. Left hand column panels in each case show unmagnified images (scale bars 50 µm). Note the lower left panels are the same as in Fig 1C. Middle and right-hand panels are x8 fold magnified views of the white boxed areas (scale bars 5 µm). Some LYVE-1 lined rings (white arrow heads) can be seen underlying sites of HA bead binding in untreated cells (top). However, these are much denser and more numerous in Lat B treated cells (middle panel) and are absent from cells incubated with LYVE-1 blocking antibody (H, right panel).

Fig. S2: Controls and outlines on the LYVE-1 mobility experiments A) Example of statistical analysis of the histograms of sFCS transit times for LYVE-1 in primary HDLECs depicting cumulative, logarithmic and linear representations of the data (from panels D and E in Figure 2) and their fit. B) Surface expression levels of LYVE-1, as a function of time (days) in post-confluent culture plotted as the median fluorescent intensity (MFI) as determined by FACS. C) Average diffusion coefficient values of LYVE-1 in HDLECs as a function of time in post-confluent culture (days) detected by sFCS. D) Box

charts of sFCS transit times comparing untreated and DMSO treated cells (10-45 minutes). No effect on the transit times of LYVE-1 was observed. E, F) Effect of labelling methods on diffusion of LYVE-1 detected by sFCS and FRAP respectively. G) Changes in LYVE-1 diffusion coefficient values with the confocal techniques FRAP, scanning FCS (sFCS) and point FCS (pFCS) determined using HDLECs transfected with GFP tagged LYVE-1. H, I) Principle of STED-FCS and the diffusion modes determined by the D(d) dependency. H) Scheme of fluorescent molecules moving through a confocal observation spot which does not allow for the disclosure of nanoscale details (left) and through a STED observation spot (right) that can identify nanoscale hindered diffusion of these molecules. I) The different diffusion modes that can be determined by tuning the observation spot from confocal to smaller diameters (d) illustrating the dependence of D(d) for free Brownian movement, hopping diffusion, domain incorporation and transient trapping or confinement as indicated. Figures are adapted from (54, 72).

Confocal FRAP on ACP tagged hLYVE-1 in HDLECs						
	D ($\mu m^2/s$)				% mobile normalised	
	D_1	SD	D_2	SD	A_1	A_2
Untreated	0.02	0.00	0.12	0.02	48	52
CK-666	0.03	0.01	0.21	0.04	39	61

Table S1: Average calculated D values of hLYVE-1 diffusion in FRAP before and after treatment with CK-666 (n=10 cells, Unpaired t-test p values: D1 = 0.0007, D2= 0.0002).

Confocal sFCS on primary HDLECs with actin depolymerisation							
Untreated		Cyto D		Lat B		CK-666	
D ($\mu m^2/s$)	SEM	D ($\mu m^2/s$)	SEM	D ($\mu m^2/s$)	SEM	D ($\mu m^2/s$)	SEM
0.15	0.01	0.16	0.01	0.18	0.02	0.16	0.01

Table S2: Average calculated D values of hLYVE-1 diffusion in sFCS before and after actin depolymerising drug treatments (n = 3, Unpaired t-tests, p values: Cyto D = 0.0186, Lat B = 0.0025, CK-666 = 0.0566).

Confocal sFCS hLYVE-1 mutants					
hLYVE-1 FL		hLYVE-1 Δ263		hLYVE-1 Δ259	
D ($\mu m^2/s$)	SEM	D ($\mu m^2/s$)	SEM	D ($\mu m^2/s$)	SEM
0.14	0.01	0.21	0.01	0.22	0.02

Table S3: Average calculated D values of hLYVE-1 cytoplasmic tail truncation mutants in sFCS (n = 3, Unpaired t-tests, p values: Δ263 = 0.0004, Δ259 = 0.0003).

Drug Treatments	Observation spot size (nm)							
	50		70		100		250	
	D ($\mu m^2/s$)	SEM	D ($\mu m^2/s$)	SEM	D ($\mu m^2/s$)	SEM	D ($\mu m^2/s$)	SEM
Untreated	0.09	0.01	0.10	0.004	0.10	0.01	0.22	0.02
CK-666	0.09	0.001	0.12	0.01	0.11	0.01	0.23	0.01
Lat B	0.09	0.001	0.12	0.01	0.12	0.01	0.25	0.02

Table S4: Calculated STED-FCS diffusion co-efficients of hLYVE-1 before and after actin depolymerising drug treatments. Average from calculated D values of 3 experimental replicates with SEM. Unpaired t-test compared to Untreated; *p* values for CK-666 at spot size 250 nm:

0.6262, 100 nm: 0.6206, 70 nm : 0.0818 and 50 nm : 0.6772; *p* values for Lat B at spot size 250 nm: 3620, 100 nm : 0.3018, 70 nm: 0.0053 and 50 nm: 0.6027.

hLYVE-1 mutants	Observation spot size (nm)									
	50		60		80		100		250	
	D ($\mu m^2/s$)	SEM	D ($\mu m^2/s$)	SEM	D ($\mu m^2/s$)	SEM	D ($\mu m^2/s$)	SEM	D ($\mu m^2/s$)	SEM
FL	0.06	0.003	0.09	0.01	0.11	0.01	0.12	0.01	0.22	0.01
Δ263	0.14	0.08	0.13	0.03	0.13	0.01	0.17	0.02	0.30	0.01
Δ259	0.14	0.03	0.16	0.02	0.16	0.02	0.19	0.00	0.35	0.02

Table S5: Calculated STED-FCS diffusion co-efficients of hLYVE-1 cytoplasmic tail truncation mutants. Average from calculated D values of 3 experimental replicates with SEM. Unpaired t-test compared to FL; *p* values for Δ263 at spot size 250 nm = 0.0021, 100 nm = 0.0497, 80 nm = 0.2705, 60 nm = 0.2162 and 50 nm = 0.4164; *p* values for Δ259 at spot size 250 nm = 0.0023, 100 nm = 0.0012, 80 nm = 0.0483, 60 nm = 0.0369 and 50 nm = 0.0732.

Fig S1

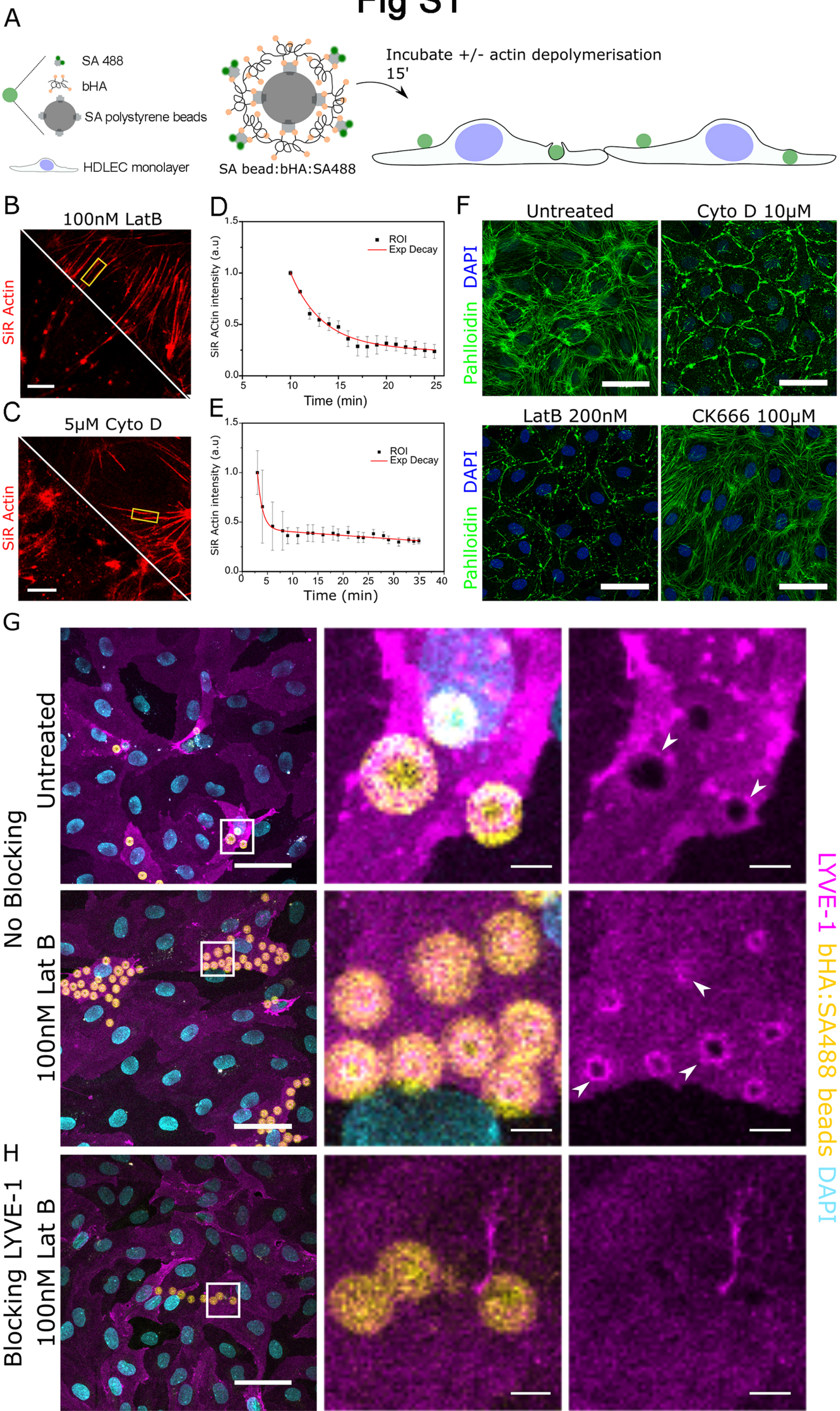

A
SA 488
bHA
SA polystyrene beads
HDLEC monolayer
SA bead:bHA:SA488
Incubate +/- actin depolymerisation
15'
B
100nM LatB
SiR Actin
C
5μM Cyto D
SiR Actin
D
ROI
Exp Decay
SiR ACtin intensity (a.u)
Time (min)
E
ROI
Exp Decay
SiR Actin intensity (a.u)
Time (min)
F
Untreated
Cyto D 10μM
LatB 200nM
CK666 100μM
Pahlloidin DAPI
G
No Blocking
Untreated
100nM Lat B
H
Blocking LYVE-1
100nM Lat B
LYVE-1 bHA:SA488 beads DAPI

Fig S2

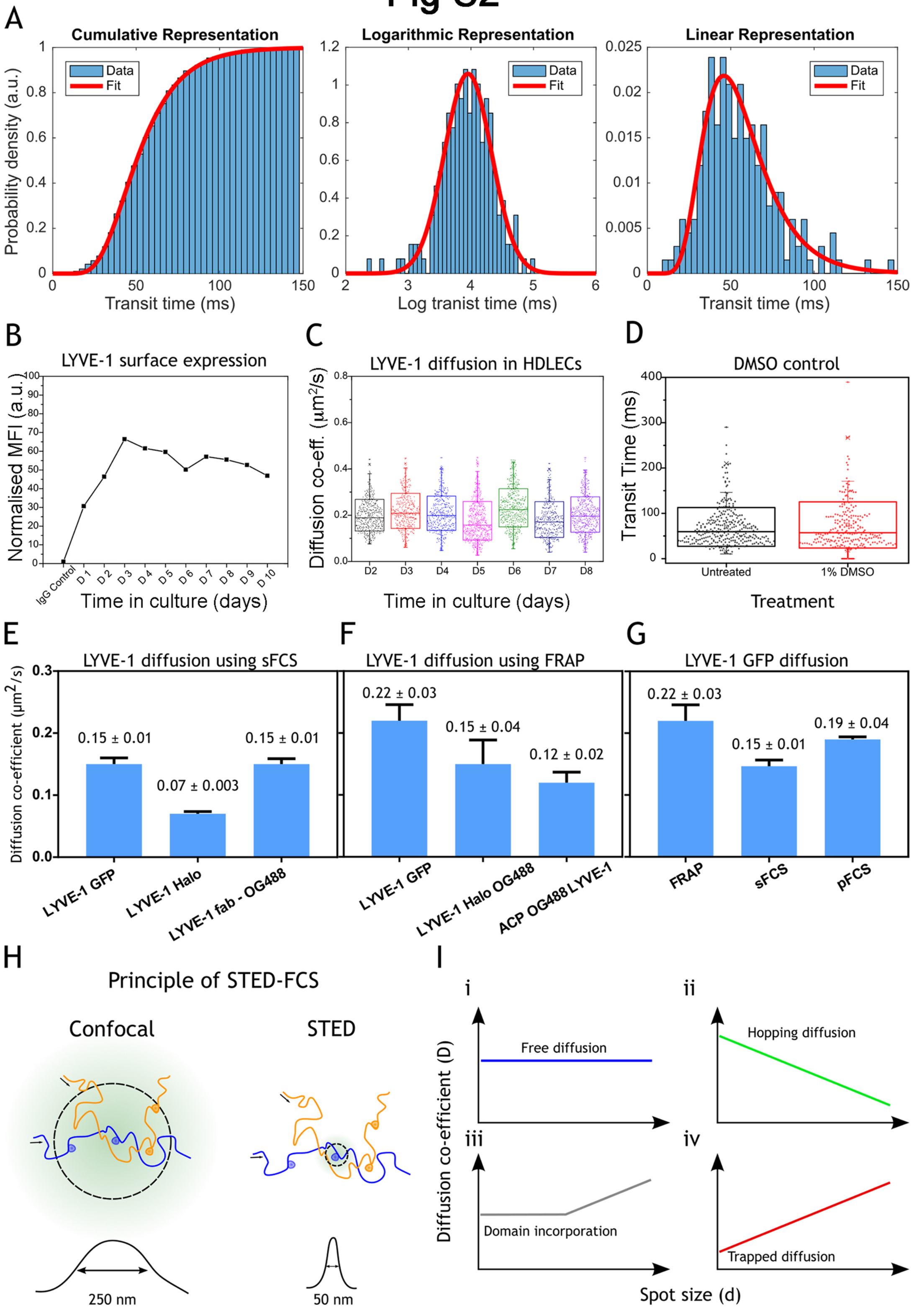
A
Cumulative Representation
Logarithmic Representation
Linear Representation
Data
Fit
Probability density (a.u.)
Transit time (ms)
Log tranist time (ms)
B
LYVE-1 surface expression
Normalised MFI (a.u.)
IgG Control
D 1
D 2
D 3
D 4
D 5
D 6
D 7
D 8
D 9
D 10
Time in culture (days)
C
LYVE-1 diffusion in HDLECs
Diffusion co-eff. (µm²/s)
D2
D3
D4
D5
D6
D7
D8
Time in culture (days)
D
DMSO control
Transit Time (ms)
Untreated
1% DMSO
Treatment
E
LYVE-1 diffusion using sFCS
Diffusion co-efficient (µm²/s)
0.15 ± 0.01
0.07 ± 0.003
0.15 ± 0.01
LYVE-1 GFP
LYVE-1 Halo
LYVE-1 fab - OG488
F
LYVE-1 diffusion using FRAP
0.22 ± 0.03
0.15 ± 0.04
0.12 ± 0.02
LYVE-1 GFP
LYVE-1 Halo OG488
ACP OG488 LYVE-1
G
LYVE-1 GFP diffusion
0.22 ± 0.03
0.15 ± 0.01
0.19 ± 0.04
FRAP
sFCS
pFCS
H
Principle of STED-FCS
Confocal
STED
250 nm
50 nm
I
i
ii
iii
iv
Free diffusion
Hopping diffusion
Domain incorporation
Trapped diffusion
Diffusion co-efficient (D)
Spot size (d)
